# Therapeutic Application of Bacteriophage PHB02 and Its Putative Depolymerase Against *Pasteurella multocida* Capsular Type A in Mice

**DOI:** 10.3389/fmicb.2018.01678

**Published:** 2018-08-07

**Authors:** Yibao Chen, Erchao Sun, Lan Yang, Jiaoyang Song, Bin Wu

**Affiliations:** ^1^State Key Laboratory of Agricultural Microbiology, College of Veterinary Medicine, Huazhong Agricultural University, Wuhan, China; ^2^The Cooperative Innovation Center for Sustainable Pig Production, Huazhong Agricultural University, Wuhan, China; ^3^Key Laboratory of Preventive Veterinary Medicine in Hubei Province, Wuhan, China

**Keywords:** Bacteriophage, *Pasteurella multocida*, mutants, receptor, putative capsule depolymerase

## Abstract

Phage PHB02 specifically infects *Pasteurella multocida* capsular serogroup A strains. In this study, we found that capsule deletion mutants were not lysed by PHB02, suggesting that the capsule of *P. multocida* serogroup A strains might be the primary receptor. Based on sequence analysis, a gene encoding a phage-associated putative depolymerase was identified. The corresponding recombinant depolymerase demonstrated specific activity against capsular serogroup A strains but did not strip capsule deletion mutants. *In vivo* experiments showed that PHB02 was retained at detectable levels in the liver, spleen, kidneys, lung, and blood, at 24 h post-administration in mice. Depolymerase plus serum significantly reduced the number of viable wild-type *P. multocida* strain HB03 cells (3.5–4.5 log decrease in colony-forming units). Moreover, treatment with phage or purified depolymerase resulted in significantly increased survival of mice infected with *P. multocida* HB03, and an absence of increase of eosinophils and basophils or other pathological changes when compared with the control group. These results show that phage PHB02 and its putative depolymerase represent a novel strategy for controlling *P. multocida* serogroup A strains.

## Introduction

Gram-negative bacterium *Pasteurella multocida* is part of the normal respiratory microbiota of many animals. However, it is potentially pathogenic in domestic and agricultural animals (e.g., cats, dogs, cattle, pigs, and rabbits) (Ewers et al., 2006; García-Alvarez et al., 2015) and in some bird species (Wilkie et al., 2012). Importantly, human *P. multocida* infections are not common and are usually associated with the older adult or immunocompromised individuals (Baillot et al., 2011; Abreu et al., 2018). *P. multocida* is generally classified into five capsular serogroups (A, B, D, E, and F) (Carter, 1955) and 16 somatic lipopolysaccharide (LPS) serotypes (1–16) (Heddleston et al., 1972). Capsular serogroup A strains are most often associated with bovine hemorrhagic septicemia and avian cholera (Chung et al., 2001; Borrathybay et al., 2003). It is estimated that the cost associated with treating bovine hemorrhagic septicemia is greater than $500 million per year in North America (Miles, 2009). In addition, some birds, such as turkeys and waterfowl, are more susceptible to serious diseases caused by capsular serogroup A strains (Samuel et al., 2005; Wilkie et al., 2012). Serogroups B and E cause hemorrhagic septicemia in cattle and buffalo (Boyce and Adler, 2000), while serogroup D is responsible for atrophic rhinitis in pigs (Davies et al., 2003), and serogroup F is usually associated with poultry cholera (Jonas et al., 2001; Jaglic et al., 2011).

Polysaccharide capsules are produced by a wide range of bacteria, and they provide protection against the host immune system (Jann and Jann, 1987; Petruzzi et al., 2017). Moreover, the capsules of many pathogenic bacteria impair phagocytosis and reduce or inhibit complement-mediated killing (Boyce and Adler, 2000; Scholl and Merril, 2005). Some reports show that loss of the capsule in *P. multocida* is associated with reduction or loss of virulence (Sthitmatee et al., 2011). For example, mutant *P. multocida* strain P-1059 (serovar A:3) was completely attenuated in chickens (Sthitmatee et al., 2011).

In recent years, the emergence of multidrug-resistant bacteria has gained much attention. Previous studies have isolated *P. multocida* strains, showing resistance to chloramphenicol, enrofloxacin, lincomycin, norfloxacin, and doxycycline-HCl, from various animals, including chicken, ducks, turkeys, quails, and geese (Shivachandra et al., 2004; Sarangi et al., 2015; Maynou et al., 2017). Therefore, there is a need to identify and develop new therapeutic strategies against these multidrug-resistant *P. multocida* strains.

Phages have been isolated from all environments in which bacteria exist. Phages demonstrate high specificity and effectiveness in killing bacterial pathogens, especially multidrug-resistant bacteria (Bhetwal et al., 2017; Reindel and Fiore, 2017; Cha et al., 2018). There are a few reports on *P. multocida* phages, with only a few temperate phage genomes studied in depth (Campoy et al., 2006). However, our previous studies have shown that phage PHB02, isolated from wastewater, is a lytic phage specific for *P. multocida* serogroup A strains (Chen et al., 2018).

Recently, there have been several reports on the successful application of lytic phages in the treatment of clinical multidrug-resistant bacterial strains (Bhetwal et al., 2017; Reindel and Fiore, 2017; Cha et al., 2018). These phages inhibited or killed the bacterial pathogens but were harmless to the animal or human host (Brunel and Guery, 2017; Cheng et al., 2018). Furthermore, phage-derived proteins, such as lysate endolysin and lyase, have been used independently for pathogen control (Drulis-Kawa et al., 2015; Benešík et al., 2018; Ha et al., 2018). There is also increasing interest in using phage depolymerases as biocontrol agents (Majkowska-Skrobek et al., 2016; Guo et al., 2017; Hsieh et al., 2017). Phage depolymerases can specifically recognize and degrade capsular polysaccharides, extracellular polysaccharides, and *O*-antigen (Corbett and Roberts, 2008). Importantly, Rakhuba et al. (2010) reported that the bacterial capsule can act as a primary receptor for phage, which often possesses tail fibers or tail spikes with capsule depolymerization activity. Previously, Scholl and Merril (2005) reported that phage K1F encodes a depolymerase that allows the phage to recognize and degrade the polysaccharide capsule of *Escherichia coli*. In addition, phages may encode several different capsule depolymerases to degrade the capsules of multiple bacterial species (Hsieh et al., 2017). Moreover, bacterial capsules can function as primary receptors for different types of phages (Hsieh et al., 2017). As such, unencapsulated strains are not infected by phages and are not degraded by phage-derived depolymerases.

Previously, we described the biological characteristics and genomic properties of phage PHB02 (Chen et al., 2018). PHB02 is specific for *P. multocida* serogroup A strains and can form plaque-surrounding halos on agar plates. This typical halo ring is associated with phage-derived depolymerases (Latka et al., 2017). Depolymerases (or phages) have proven to be effective for the prevention or eradication of biofilms (Gutiérrez et al., 2016; Guo et al., 2017), and treatment with recombinant depolymerase derived from phage NTUH-K2044-K1-1 resulted in significantly increased survival of mice infected with capsular type K1 *Klebsiella pneumoniae* (Lin et al., 2014). Based on these results, we hypothesized that depolymerases may have considerable potential as biocontrol agents.

In this study, we isolated putative depolymerase Dep-ORF8 from *P. multocida* phage PHB02 and demonstrated that neither the phage nor the isolated putative depolymerase had activity against a capsule-minus *P. multocida* HB03 deletion mutant strain. We also investigated the effects of phage PHB02 or putative depolymerase Dep-ORF8 treatment in mice infected with *P. multocida*.

## Materials and Methods

### Bacterial Strains and Animals

*Pasteurella multocida* strains were grown at 37°C in tryptic soy broth (TSB; Becton, Dickinson and Company, Glencoe, MD, United States) or tryptic soy agar (TSA; Becton, Dickinson and Company, Glencoe, MD, United States) medium supplemented with 10% (vol/vol) sterile, defibrinated sheep blood (Jiulongbio, Zhengzhou, China). Soft agar was prepared by the addition of 0.75% (wt/vol) agar to TSB medium. All *P. multocida* strains used in this study are listed in Supplementary Table S1. *Escherichia coli* BL21 (DE3) was cultured in Luria-Bertani broth (LB; 10 g tryptone, 5 g yeast extract, 10 g NaCl per liter) at 37°C. If required, antibiotics were added to the medium (kanamycin, 50 μg/ml; ampicillin 100 μg/ml).

A capsule-deficient *hexA*-knockout mutant was generated from wild-type *P. multocida* strain HB03 by the method described by Tabatabaei et al. (2002). Briefly, forward and reverse primers were designed from the *hexA* sequence of *P. multocida* HB03 (GenBank accession number CP003328; Supplementary Table S2). The *hexA* fragments were removed as *Sal*I/*Eco*RI- and *Eco*RI/*Sac*I-digested fragments and cloned into the suicide vector pBC-SK that was cut with the same enzymes. The kanamycin resistance cassette amplified from plasmid pUC-4k digested with *Eco*RI was inserted into pBC-SK to generate *hexA*-Km-pBC-SK. Plasmid *hexA*-Km-pBC-SK was inserted into *P. multocida* HB03 by electroporation (Zhao et al., 2016).

Five-week-old female BALB/c mice (20–25 g in weight) were purchased from the Experimental Animal Centre of Huazhong Agricultural University, Wuhan, China. All animal procedures were performed in strict accordance with the Regulations for the Administration of Affairs Concerning Experimental Animals, approved through the State Council of the People’s Republic of China (1988.11.1), and with the approval of the Animal Welfare and Research Ethics Committee at Huazhong Agricultural University.

### Identification and Analysis of Putative Depolymerase-Encoding Genes

The complete genome sequence of phage PHB02 (GenBank accession number MF034659) has been described earlier (Chen et al., 2018). The Position-Specific Iterative Basic Local Alignment Search Tool^1^ was used to identify putative depolymerase-encoding genes in the PHB02 genome, and Phyre2^2^ was used to predict the structure of the depolymerase (Kelley et al., 2015).

### Expression and Purification of the Phage-Derived Putative Depolymerase

Phage PHB02 open reading frame 8 (*orf* 8) was amplified by polymerase chain reaction using primers A2-*Kpn*I (5′-CGGGGTACCGTGGAGTTTCTGATTCCCTT-3′) and A2-*Bam*HI (5′-CGGGATCCTCATGCTAGCTTCTTTGTCTT-3′). The purified amplicon was cloned into plasmid pCold TF, generating pCold-ORF8, which was then transformed into *E. coli* BL21(DE3). The recombinant His-tagged PHB02-ORF8 protein was expressed by induction with 0.1 mM isopropyl β-D-1-thiogalactopyranosid for 18 h at 16°C and was then purified as described by Guo et al. (2017). Briefly, PHB02-ORF8 was purified from the soluble fraction using a Ni-nitrilotriacetic acid column (Genscript, Wuhan, China) before being eluted and dialyzed overnight at 4°C against a 1000-fold volume of phosphate-buffered saline (PBS) buffer (137 mM NaCl, 2.7 mM KCl, 10 mM Na_2_HPO_4_, 1.8 mM KH_2_PO_4_, pH = 7.4). The hexa-histidine tag was then removed from the purified protein by digestion with thrombin (Solarbio, Shanghai, China). The purified protein solution was again passed through the column and then concentrated to 0.36 μg/μl by centrifugation over a 30-kDa ultrafiltration tube (Solarbio, Shanghai, China). The resulting purified PHB02-ORF8 protein was analyzed by sodium dodecyl sulfate polyacrylamide gel electrophoresis as described by Guo et al. (2017). Different doses were obtained by dilution of the stock depolymerase, which was stored at −80°C.

### Spot Test

Depolymerase Dep-ORF8 activity against host strains was determined by spot test, as described by Guo et al. (2017). Briefly, molten soft TSA (0.75% agar) supplemented with 10% (vol/vol) sterile defibrinated sheep blood was inoculated with 300 μl of the host bacterial strain grown to exponential phase. This mixture was then poured onto the surface of a TSA (1.5% agar) plate supplemented with 10% (vol/vol) sterile defibrinated sheep blood and allowed to set. After drying, aliquots of a serial dilution of purified PHB02-ORF8 protein (360 to 1.4 ng) were spotted onto the surface of the double-layer agar plates. Elution buffer diluted in PBS was used as a control. To determine the specificity of the Dep-ORF8 depolymerase, capsular type D or F *P. multocida* strains were used. Briefly, 5-μl aliquots of PHB02-ORF8 protein (360 ng) were spotted onto double-layer agar plates as described above. The plates were observed for the formation of semi-clear spots for 6 h at 37°C. This experiment was repeated three times.

### Phage Killing Assay

*In vitro* phage killing assays against the *P. multocida* HB03 strains were conducted as described by Dalmasso et al. (2016). Briefly, phage PHB02 was added to the *P. multocida* HB03 strains or capsule mutant strains (10^5^ colony-forming units (CFU)/ml) at a multiplicity of infection of 10 or 100, and then cultured at 37°C for 30 or 60 min, respectively. Bacterial counts were determined by plating serial dilutions of the cultures. This experiment was repeated three times. The experimental data were analyzed by using a two-way analysis of variance. The data obtained are expressed as the mean ± standard deviation (SD).

### Serum Sensitivity Assay

Serum sensitivity was determined as previously described (Lin et al., 2017). Briefly, exponentially growing bacteria [1–3 × 10^7^ colony-forming units (CFU)/ml] mixed with depolymerase (100 μg/ml) were incubated with different sera, including 75% mouse serum (Hengyuan, Shanghai, China), 75% human serum (Hengyuan, Shanghai, China), heat-inactivated mouse or human serum (56°C, 30 min), 75% mouse whole blood (Hengyuan, Shanghai, China), and PBS (as a control) at 37°C for 3 h. Bacterial counts were determined by plating serial dilutions of the cultures. Experimental data were analyzed by one-way analysis of variance. The data obtained are expressed as the mean ± SD.

### Acute Toxicity Assay

To study the toxicity of the phage, nine 5-week-old female BALB/c mice were randomly divided into three groups. The groups were inoculated intraperitoneally with 100 μl of phage [10^9^ plaque-forming units (PFU)/ml], 100 μl of depolymerase Dep-ORF8 (36 μg), or an equal volume of PBS buffer. The mice were then observed daily for 7 days.

Health scores were determined as previously described (Zhang et al., 2016). Briefly, the health status of each group of mice was given a score between zero and five, as follows: five: normal health, condition unremarkable; four: decreased physical activity and ruffled fur; three: lethargy and hunched back; two: exudative accumulation around partially closed eyes; one: near death; zero: dead. The total score for each group was recorded at least three times per day. The mice were euthanized by CO_2_ at 7 days post-inoculation and subjected to histopathological examination. The liver, spleen, kidney, and lungs were removed and immediately placed in 4% formalin, then dehydrated with different concentrations of alcohol, and treated with wintergreen oil overnight. Paraffin-embedded tissue was cut into slices (∼5 μm thick) with a microtome (ZENDA, United States). Samples were dewaxed and then stained with hematoxylin and eosin or toluidine blue, as described previously (Zhang et al., 2016). The data describing the health status of each group of mice are expressed as the mean ± SD.

### Phage *in vivo* Distribution Test

To examine phage distribution patterns in mice, 15 5-week-old female BALB/c mice were randomly divided into three groups. The groups were inoculated intraperitoneally with 100 μl of phage PHB02 (10^9^ pfu/ml). Individual mice were then euthanized at 6, 12, 24, 48, and 72 h post-inoculation. The amount of phage present in the liver, lungs, kidney, and spleen was measured and reported as PFU/g of tissue, while blood counts were measured and reported as PFU/ml. The titration of phage particles was conducted via the double-layer agar method. The data are expressed as the mean ± SD.

### Mouse Infection and Treatment

To examine the protective effects of phage and capsule depolymerase therapy, the minimum lethal dose (MLD) of wild-type *P. multocida* HB03 was first determined as described previously (Xia et al., 2015). Briefly, 15 5-week-old female BALB/c mice were randomly divided into five groups and inoculated intraperitoneally with 100 μl of different doses of *P. multocida* (10 CFU, 20 CFU, 40 CFU, 80 CFU, and 160 CFU, respectively). Once the MLD was determined, 2× the MLD was used as the challenge dose. Forty-eight 5-week-old female BALB/c mice were then randomly divided into eight groups (a–h). Groups a–g were inoculated intraperitoneally with 100-μl doses of 80 CFU of wild-type *P. multocida* HB03, while the control group h animals were inoculated intraperitoneally with an equivalent volume of PBS. Groups b and c were then inoculated intraperitoneally with 100 μl of phage PHB02 (10^9^ pfu/ml) at 6 h and 12 h post-bacterial inoculation, while group d mice were inoculated intraperitoneally with 100 μl of phage PHB02 (10^9^ pfu/ml) at 12 h following bacterial inoculation, and then once daily for 5 days. Similarly, groups e and f were inoculated intraperitoneally with 100 μl of depolymerase Dep-ORF8 (36 μg) at 6 h and 12 h post-bacterial inoculation, whereas group g mice were inoculated intraperitoneally with 100 μl of depolymerase Dep-ORF8 (36 μg) at 12 h after bacterial inoculation, and then once daily for 5 days. The mice were then observed daily for 21 days. The data pertaining to the health status of each group of mice are expressed as the mean ± SD. Survival was analyzed by Kaplan–Meier analysis with a log-rank test (statistically significant at *P* < 0.05).

## Results

### Analysis of Putative Depolymerase-Encoding Genes

In a previous study, we observed that phage PHB02 produced halos around the plaques, which we suspected was related to a putative phage-derived depolymerase. As phage depolymerases are usually part of the tail spike or tail fiber, we hypothesized that the tail fiber of phage PHB02, encoded by *orf8*, might be related to phage depolymerase. We therefore used Phyre2 to predict the secondary structure of Dep-ORF8, which consisted of 24 α-helices and 47 β-structures, as well as disordered regions. In addition, the model of Dep-ORF8 exhibited limited similarity to other known proteins but showed a confidence value of 97.8% (13% coverage) with homologous protein Clp9Ha (Supplementary Figure S1).

### Expression and Purification of the Phage Depolymerase

**FIGURE 1:**
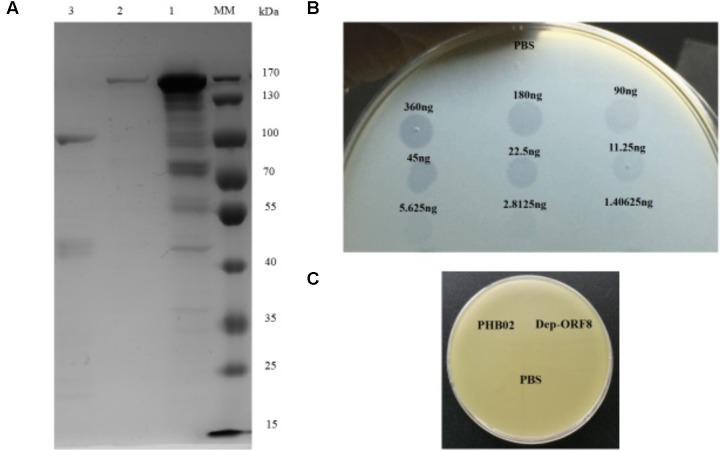
Dep-ORF8 overexpression and activity. **(A)** Sodium dodecyl sulfate polyacrylamide gel electrophoresis analysis of purified Dep-ORF8. Lane MM, protein marker; lane 1, 20 μg of supernatant from the induced *E. coli* BL21(DE3) cells; lane 2, 200 ng of purified Dep-ORF8 (containing the his-tag); lane 3, 200 ng of purified Dep-ORF8 (minus the his-tag). **(B)** Activity of depolymerase Dep-ORF8 against wild-type *P. multocida* strain HB03. Serial dilutions of Dep-ORF8 were spotted onto the plate containing *P. multocida* HB03. Elution buffer diluted in PBS was used as a control. **(C)** Spot tests examining the ability of phage PHB02 and depolymerase Dep-ORF8 to lyse the capsule-deficient mutant strain. Aliquots (5 μl) of phage PHB02 at a concentration of 10^8^ pfu/ml and depolymerase Dep-ORF8 (360 ng) were spotted onto a plate containing the mutant strains. The plates were observed for 6 h at 37°C.

Dep-ORF8 was predicted to be 109.6 kDa in size and, following purification, the concentration of the recombinant protein was 0.36 μg/μl (**Figure 1A**). Spot tests showed that at amounts ≥ 2.8125 ng, purified recombinant Dep-ORF8 produced a translucent spot in the lawn of host bacteria (**Figure 1B**). The assay indicated that all 31 *P. multocida* capsular type A strains were sensitive to the recombinant Dep-ORF8 protein (Supplementary Table S1). Neither the phage nor the purified Dep-ORF8 protein hydrolyzed the capsule-deficient mutant strains (**Figure 1C**).

### Phage Killing Assay

**FIGURE 2:**
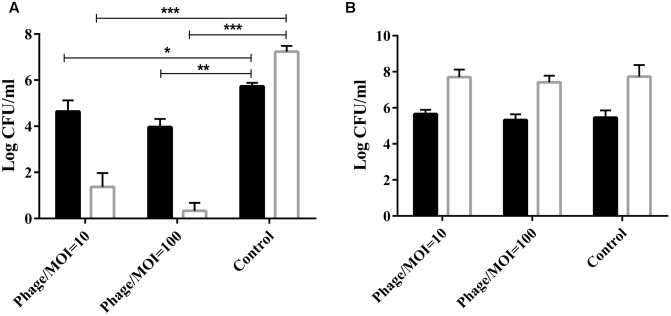
*In vitro* activity of the phage against the wild-type and capsule-deficient mutant strains. **(A)** *In vitro* activity of the phage against wild-type *P. multocida* strain HB03. The phage was mixed with the bacteria at a MOI of 10 or 100 and then cultured at 37°C for 30 min (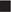) or 60 min (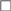). Results show the number of viable bacteria following incubation. Experimental data were analyzed by two-way analysis of variance and are expressed as the mean ± SD. The viable cell counts in each of the treatment groups were compared with that of the control, with significant differences indicated by ^∗^*P* < 0.05; ^∗∗^*P* < 0.01; and ^∗∗∗^*P* < 0.001. **(B)** *In vitro* activity of the phage against the capsule-deficient *P. multocida* strain. Conditions and data interpretation were the same as described in (A).

The phage was co-cultured with *P. multocida* HB03 for 1 h at a multiplicity of infection (MOI) of 10 or 100. Results showed that at a MOI of 100, phage PHB02 significantly reduced the number of viable wild-type HB03 cells when compared with the control treatment after 60 min of incubation (*P* < 0.01) (**Figure 2A**). The phage had no effect on the survival of the capsule-mutant strains (**Figure 2B**).

### Serum Sensitivity Assay

**FIGURE 3:**
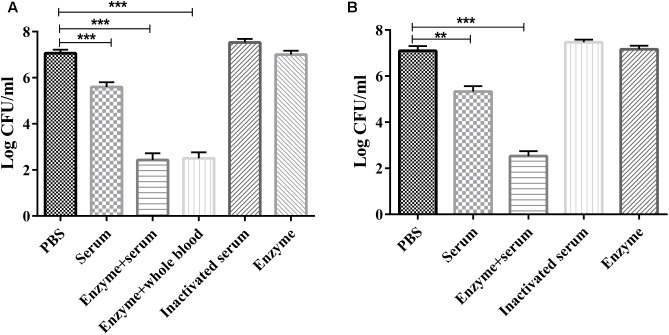
Serum sensitivity assay. **(A)** *P. multocida* strain HB03 mixed with depolymerase was incubated with mouse serum or whole-blood. **(B)** *P. multocida* strain HB03 with depolymerase was incubated with human serum. The mixtures were incubated at 37°C for 2 h. The number of viable bacteria was determined by plating and enumeration of serial dilutions of the suspension. Assays were repeated at least three times. Experimental data were analyzed by one-way analysis of variance and are expressed as the mean ± SD. The viable cell counts in each of the treatment groups were compared with that of the PBS control, with significant differences indicated by ^∗^*P* < 0.05; ^∗∗^*P* < 0.01; and ^∗∗∗^*P* < 0.001.

The serum sensitivity assay showed that purified Dep-ORF8 alone had no significant effect on bacterial survival (**Figures 3A,B**). Mouse serum, mouse whole-blood, and human serum alone had a small bactericidal effect, with a 1.2–1.7 log CFU decrease in viable cell counts. Enzyme plus serum significantly reduced the number of viable wild-type *P. multocida* strain HB03 cells (3.5–4.5 log CFU decrease). There was no significant difference in viable cell counts between the enzyme plus mouse serum group and the enzyme plus mouse whole-blood group (**Figures 3A,B**). Meanwhile, heat-inactivated serum slightly increased bacterial survival (**Figures 3A,B**). Depolymerase alone had no effect on capsule-mutant HB03 strains, while serum alone or mouse whole-blood alone killed more than 95% of capsule-mutant bacterial cells within 2 h (data not shown).

### Acute Toxicity

**FIGURE 4:**
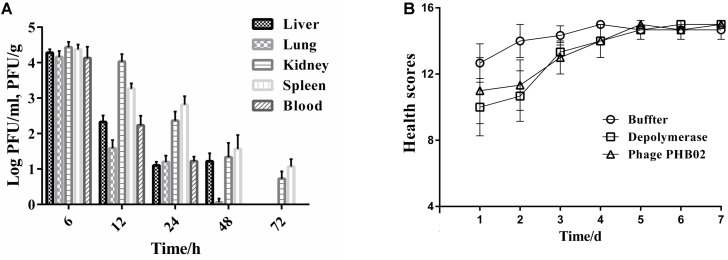
*In vivo* assays. **(A)** Distribution of phage PHB02 in mice. The experiment was repeated three times and results are expressed as the mean ± SD. **(B)** Scores of clinical symptoms post-challenge. The health status of each group of mice was given a score between zero and five, as follows: five: normal health, condition unremarkable; four: decreased physical activity and ruffled fur; three: lethargy and hunched back; two: exudative accumulation around partially closed eyes; one: near death; zero: dead. The total score for each group was recorded at least three times per day. The data are expressed as the mean ± SD.

**FIGURE 5:**
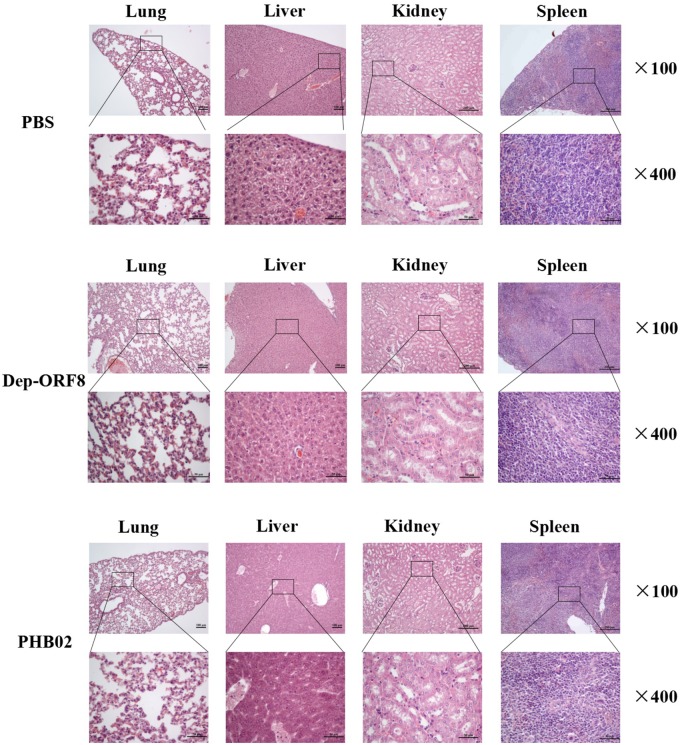
Pathological changes and histopathology of the mice challenged with phage and depolymerase. The liver, spleen, kidney, and lungs from the mice challenged with phage and depolymerase were removed and immediately placed in 4% formalin. Tissue sections were stained with hematoxylin and eosin or toluidine blue and observed under 100× and 400× magnification.

The distribution of phage in mice was studied following intraperitoneal injection of PHB02 at a dose of 1.0 × 10^8^ PFU. Phage was detected in the liver, spleen, kidney, and lungs, but was absent from blood at 48 h following phage inoculation. At 72 h post-inoculation, the phage was only detected in the spleen and kidney (**Figure 4A**). The appearance and behavior of mice was examined daily for 7 days following PHB02 inoculation. We did not observe any behavioral changes in the mice following inoculation (**Figure 4B**). More importantly, none of the tissues isolated from mice immunized with phage PHB02 or Dep-ORF8 showed an increase in eosinophils or basophils or other pathological changes when compared with the control group (**Figure 5**).

### Phage and Capsule Depolymerase Therapy

**FIGURE 6:**
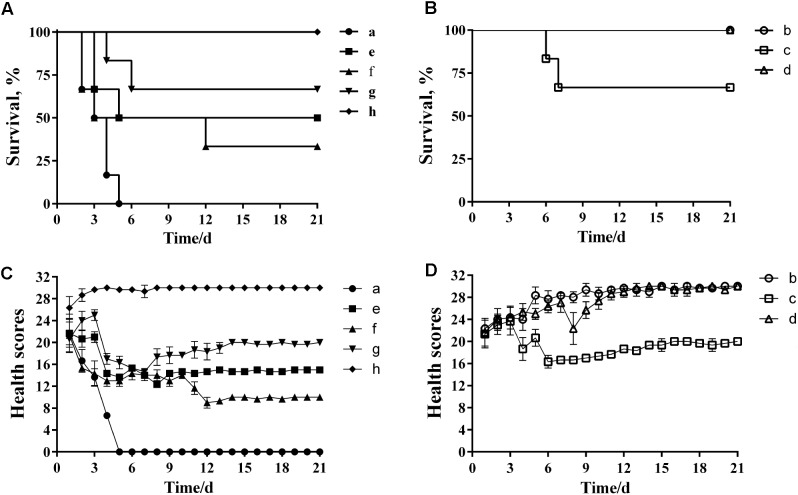
Protective effects of depolymerase and phage in mice challenged with wild-type *P. multocida* strain HB03. **(A)** Treatment efficacy of purified recombinant depolymerase. **(B)** Treatment efficacy of phage PHB02. **(C)** Clinical symptom scores post-challenge with purified recombinant depolymerase. **(D)** Clinical symptom scores post-challenge with phage PHB02. Treatment groups were as follows: a, infected with *P. multocida* strain HB03; b, phage treatment 6 h post-infection; c, phage treatment 12 h post-infection; d, multiple-dose phage treatment 12 h post-infection; e, Dep-ORF8 treatment 6 h post-infection; f, Dep-ORF8 treatment 12 h post-infection; g, multiple-dose Dep-ORF8 treatment 12 h post-infection; h, inoculated with PBS only. The total score for the health status of each group was recorded at least three times per day. The data are expressed as the mean ± SD.

Analysis of the MLD of wild-type *P. multocida* showed that more than 66% of mice died when inoculated with a bacterial dose of 40–160 CFU (Supplementary Table S3). Therefore, we concluded that the MLD of *P. multocida* HB03 in mice was 40 CFU, and accordingly used 80 CFU (2× MLD) as the challenge dose. The efficacy of depolymerase and phage treatment was then evaluated in mice infected with *P. multocida* HB03. This depolymerase therapy study showed that the survival rate of group e mice (treated with Dep-ORF8 at 6 h post-bacterial infection) was significantly increased when compared with that of control group a (*P* = 0.048), whereas the survival rate of group f (treated with Dep-ORF8 at 12 h post-bacterial infection) was not significantly increased (*P* = 0.18) (**Figure 6A**). However, mice treated with purified depolymerase at 12 h post-bacterial infection, and then daily for 5 days (group g), showed a significantly increased survival rate when compared with control group a (*P* = 0.0029). No deaths were recorded for PBS control group h (**Figure 6A**). The study also showed that none of the mice in treatment groups b or d died, while two mice from treatment group c died during the course of the experiment (on days 6 and 7, respectively) (**Figure 6B**). Treatment groups b and d had significantly greater survival than treatment group a (mice infected with HB03 alone) (*P* = 0.0006) (**Figure 6B**). No changes in behavior were observed in the 12 days following infection, after which point no further deaths occurred (**Figures 6C,D**).

## Discussion

*Pasteurella multocida* phage were first reported by Kirchner and Eisenstark (1956); however, there have since been very few reports regarding lytic phages in *P. multocida*. To our knowledge, this is the first study to demonstrate that capsular material in *P. multocida* serogroup A strains acts as a receptor for *P. multocida* phage, and that the putative depolymerase from phage PHB02 can specifically degrade capsular serogroup A strains.

The interaction of the phage with the bacterial surface is the first step in the infection process. When infecting a bacterium, the phage first needs to interact with the bacterial surface (Leiman et al., 2007; Leiman and Molineux, 2008). Polysaccharides on the cell surface normally act as a physical barrier to prevent the phage from entering the bacterial cell. However, the polysaccharides also act as primary receptors for phages, along with other protruding cell wall structures such as pili and flagella (Leiman et al., 2007; Leiman and Molineux, 2008; Li et al., 2016; Latka et al., 2017). For example, *Caudovirales* phages can recognize and attach to the bacterial surface by specifically digesting the polysaccharides on the surface via the tail-associated protein (Yan et al., 2014). Therefore, the tail apparatus of *Caudovirales* phages plays an important role in the infection of host cells. Previous research shows that the polysaccharide-degrading activity of a phage depolymerase is related to the tail structure (Scholl and Merril, 2005; Lai et al., 2016; Hsieh et al., 2017). To further verify the “primary receptor” function of polysaccharides on the bacterial cell surface, phage PHB02 was used in the current study to infect capsule-mutant strain *P. multocida* HB03. The results showed that the phage is ineffective against the capsule-mutant strain, indicating that polysaccharides are required for phage infection.

Phage PHB02 appears to be specific for capsular serotype A *P. multocida* strains, lysing 30 out of 31 tested isolates in the current study. In addition, PHB02 produces a halo around the plaque but has a narrow host range. Similar phage specificity has been reported in *K. pneumoniae* (Lin et al., 2014; Volozhantsev et al., 2016; Hsieh et al., 2017) and *E. coli* (Scholl and Merril, 2005; Leiman et al., 2007; Guo et al., 2017). Phages specifically recognize the polysaccharides of their host bacteria, and this specificity can be used for bacterial typing in epidemiological studies. In addition, phage typing can quickly and reliably identify capsular antigens. In the current study, putative depolymerase Dep-ORF8 showed activity against all 31 strains tested, suggesting that using Dep-ORF8 for capsular typing may provide a more consistent result than phage alone. In addition, depolymerase can be used as a research tool to help determine polysaccharide structure and can even be used for industrial production of complex carbohydrates.

After the bacterial capsule is stripped by depolymerase, cells are more susceptible to attack by host immune defenses (Mushtaq et al., 2004; Finlay and McFadden, 2006; Lin et al., 2014). In addition, research shows that serum can kill bacteria (Mushtaq et al., 2004). Therefore, combining depolymerase and serum may kill more bacteria than each treatment individually. To investigate this, we compared the bactericidal effects of depolymerase plus mouse serum and depolymerase plus mouse whole-blood. The results showed that there was no significant difference between the two groups. Although four of the six mice in group g survived in the *in vivo* assays (mice were treated with 100 μl of Dep-ORF8 (36 μg) at 12 h after bacterial inoculation and then once daily for 5 days), optimizing the depolymerase concentration could further increase the survival rate. Normally, bacterial infection with simultaneous treatment is important for the rescue of mice. However, to mimic a clinical treatment situation, we administered phage or depolymerase at least 6 h after bacterial infection. The results show that simultaneous treatment with phage or depolymerase is not necessary for survival following *P. multocida* infection. Therefore, it is feasible to administer phage or depolymerase at some time after initial infection.

In addition to the well-known antibacterial actions, phages are also able to mediate some anti-inflammatory activities (Jończyk-Matysiak et al., 2017; Miȩdzybrodzki et al., 2017). Therefore, the levels of inflammatory cytokines can be used as markers for monitoring treatment efficacy. Zimecki et al. (2009) demonstrated that the application of phage resulted in a down-regulation in the levels of proinflammatory cytokines (particularly TNF-α) in mice infected with *Staphylococcus aureus*. Chadha et al. (2017) showed that treatment with a phage cocktail resulted in a significant reduction of inflammatory cytokine levels, including IL-1β and TNF-α, when compared with the infection control group in a model of *K. pneumoniae*-mediated burn wound infection. Therefore, future work should be conducted to examine host inflammatory responses following phage inoculation, such as measuring the induction of IL-6 and TNF-α. Moreover, although phage specifically kill bacteria, biosafety issues cannot be ignored. Temperate phage genomes usually contain many genes with unknown functions, some of which may be virulence or resistance genes, which are difficult to predict using the limited number of sequences in the databases. In contrast, purified recombinant depolymerase can only degrade bacterial capsule, thus making it a much safer option for use *in vivo*. Application of the purified protein would strip the natural protective layer from the bacterial cells, making them much more susceptible to killing by conventional therapies both *in vitro* and *in vivo*.

## Author Contributions

YC drafted the main manuscript and performed the data analysis. YC, ES, LY, and JS planned and performed experiments. YC and BW were responsible for experimental design.

## Conflict of Interest Statement

The authors declare that the research was conducted in the absence of any commercial or financial relationships that could be construed as a potential conflict of interest.

## Footnotes

1 http://www.ncbi.nlm.nih.gov/BLAST/

2 http://www.sbg.bio.ic.ac.uk/phyre2
