# Supplementary material for: Therapeutic Application of Bacteriophage PHB02 and Its Putative Depolymerase Against Pasteurella multocida Capsular Type A in Mice
Source: Front Microbiol. 2018 Aug 7;9:1678. doi: 10.3389/fmicb.2018.01678 (PMC6090149; doi:10.3389/fmicb.2018.01678)
Supplement: Supplementary file 1 [file Image_1.PDF]

Supplementary Figure 1 Secondary structure and disorder predicted by Phyre2. "?" represents the predicted disordered regions, green helices indicate α-helices, blue arrows indicate β-strands, and faint lines indicate coils. The different colours in the "SS confidence" line represent the confidence in the prediction: red is high confidence, while blue is low confidence.

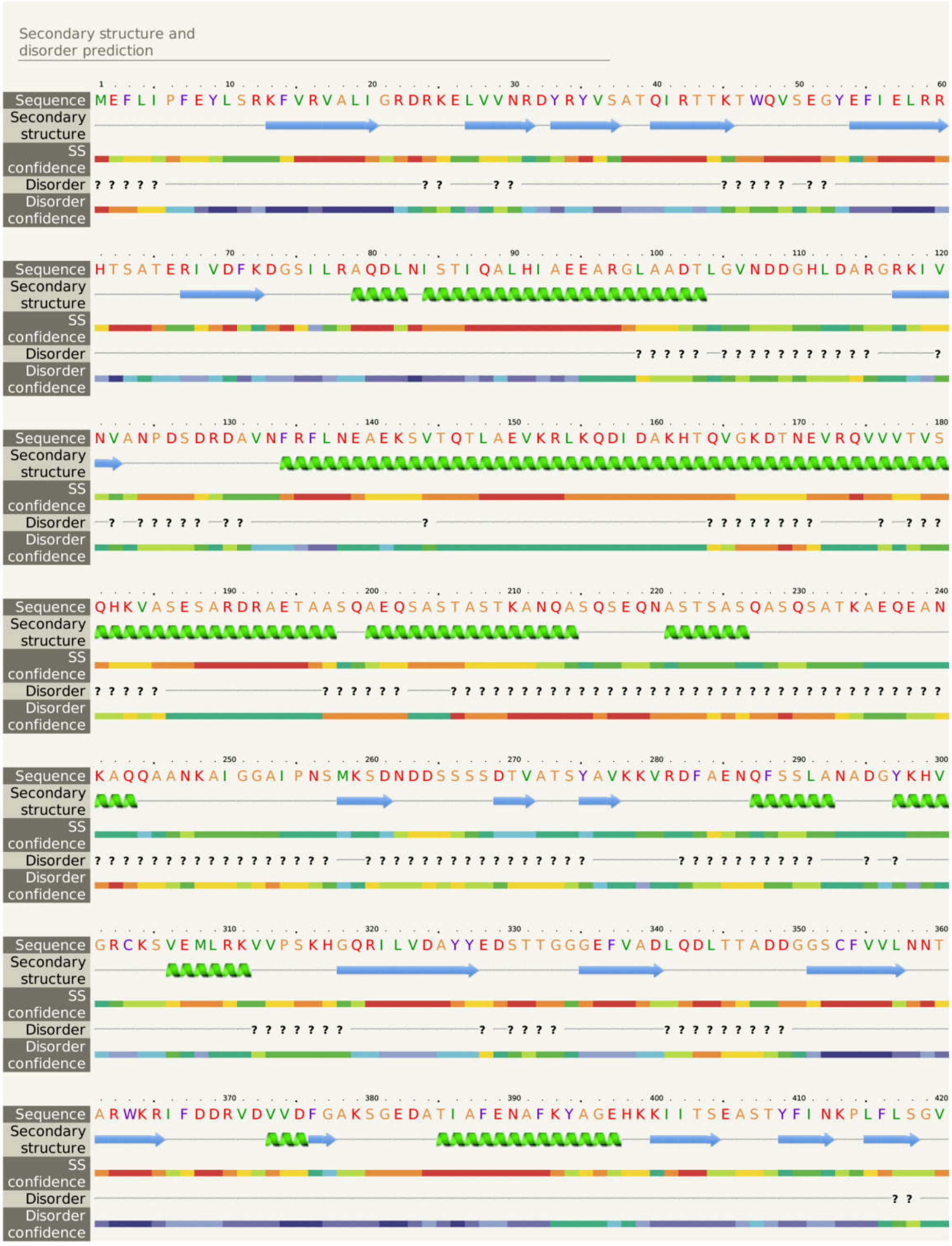

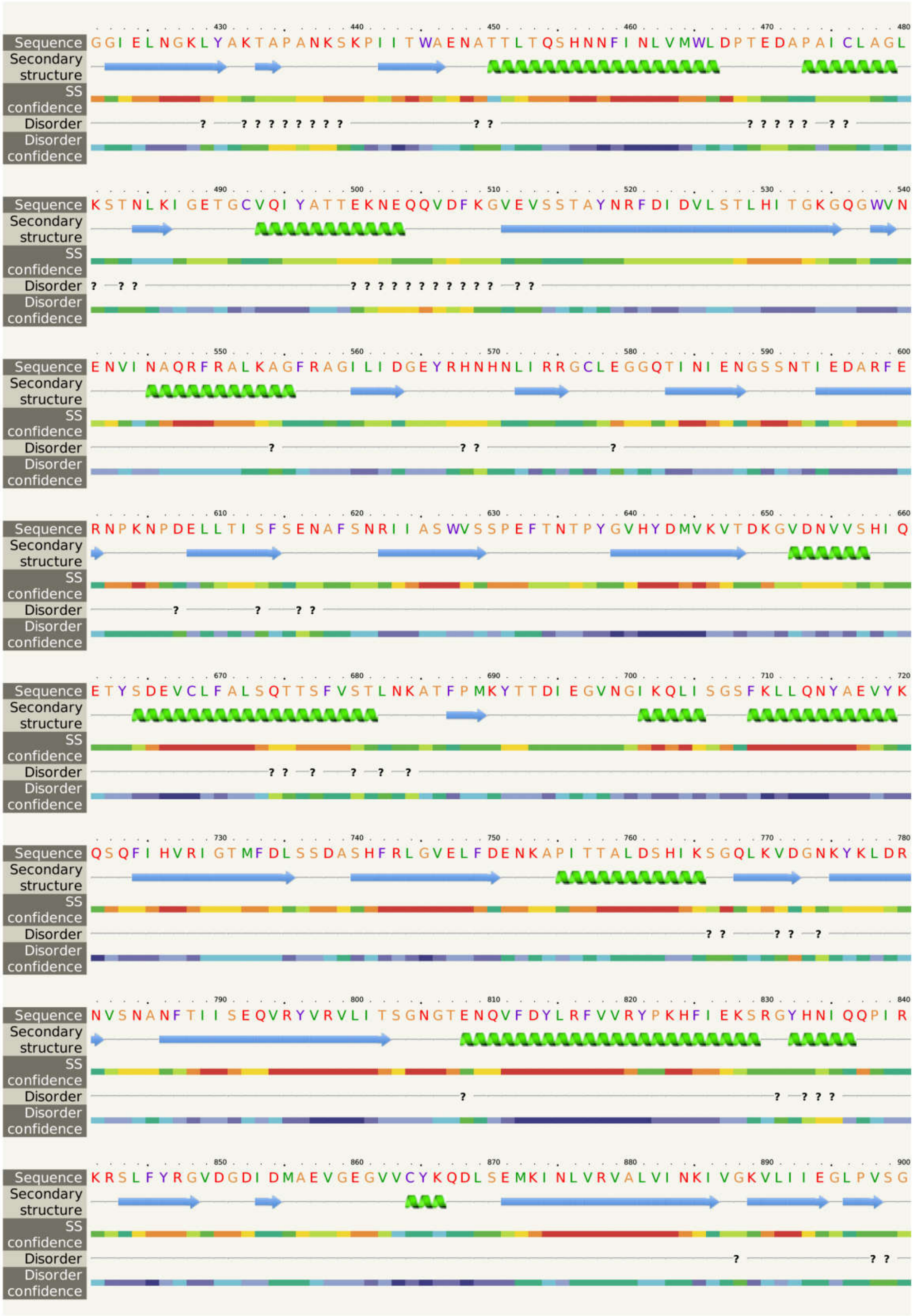

430 440 450 460 470 480
Sequence GGIELNGKLYAKTAPANKSKPIITWAENATTLTQSHNNFINLVMWLDPTEDAPAICLAGL
Secondary structure
SS confidence
Disorder
Disorder confidence
490 500 510 520 530 540
Sequence KSTNLKIGETGCVQIYATTEKNEQQVDFKGVEVSSTAYNRFDIDVLSTLHITGKGQGWVN
Secondary structure
SS confidence
Disorder
Disorder confidence
550 560 570 580 590 600
Sequence ENVINAQRFRALKAGFRAGILIDGEYRHNHNLIRRGCLEGGQTINIENGSSNTIEDARFE
Secondary structure
SS confidence
Disorder
Disorder confidence
610 620 630 640 650 660
Sequence RNPKNPDELLTISFSENAFSNRIIASWVSSPEFTNTPYGVHYDMVKVTDKGVDNVVSHIQ
Secondary structure
SS confidence
Disorder
Disorder confidence
670 680 690 700 710 720
Sequence ETYSDEVCLFALSQTTSFVSTLNKATFPMKYTTDIEGVNGIKQLISGSFKLLQNYAEVYK
Secondary structure
SS confidence
Disorder
Disorder confidence
730 740 750 760 770 780
Sequence QSQFIHVRIGTMFDLSSDASHFRLGVELFDENKAPITTALDSHIKSGQLKVDGNKYKLDR
Secondary structure
SS confidence
Disorder
Disorder confidence
790 800 810 820 830 840
Sequence NVSNANFTIISEQVRYVRVLITSGNGTENQVFDYLRFVVRYPKHFIEKSRGYHNIQQPIR
Secondary structure
SS confidence
Disorder
Disorder confidence
850 860 870 880 890 900
Sequence KRSLFYRGVDGDIDMAEVGEGVVCYKQDLSEMKINLVRVALVINKIVGKVLIIEGLPVSG
Secondary structure
SS confidence
Disorder
Disorder confidence

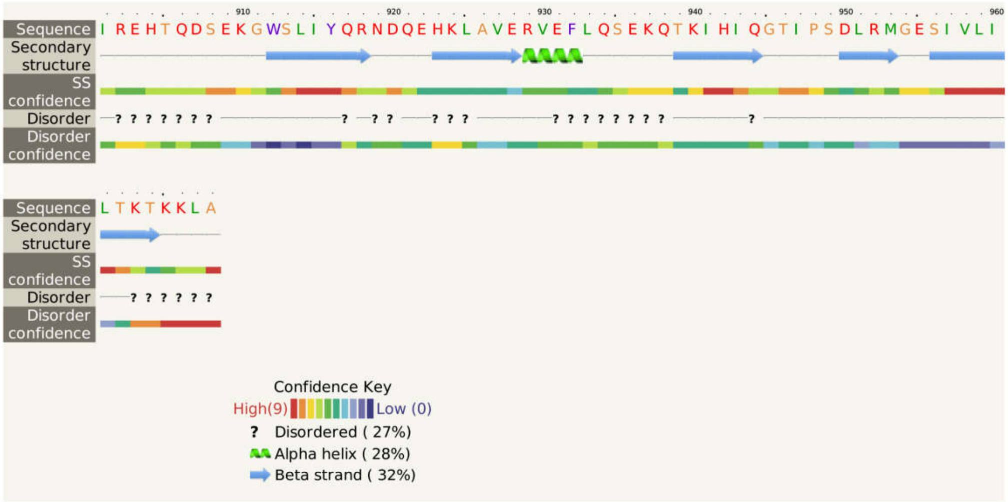

Sequence
Secondary structure
SS confidence
Disorder
Disorder confidence
910
920
930
940
950
960
I R E H T Q D S E K G W S L I Y Q R N D Q E H K L A V E R V E F L Q S E K Q T K I H I Q G T I P S D L R M G E S I V L I
L T K T K K L A
Confidence Key
High(9)
Low (0)
Disordered (27%)
Alpha helix (28%)
Beta strand (32%)
