# Supplementary material for: Therapeutic Application of Bacteriophage PHB02 and Its Putative Depolymerase Against Pasteurella multocida Capsular Type A in Mice
Source: Front Microbiol. 2018 Aug 7;9:1678. doi: 10.3389/fmicb.2018.01678 (PMC6090149; doi:10.3389/fmicb.2018.01678)
Supplement: Supplementary file 2 [file Table_1.DOCX]

**Table S1. Lytic ability of phage PHB02 and depolymerase Dep-ORF8.**

| Strain (genotype) | Origin | Isolated location | Lytic ability | |
| --- | --- | --- | --- | --- |
|  |  |  | Phage PHB02^a^ | Depolymerase^b^ |
| *Pasteurella multocida* A | Lungs | Hubei | + | + |
| *Pasteurella multocida* A | Lungs | Hunan | + | + |
| *Pasteurella multocida* A | Lungs | Hubei | + | + |
| *Pasteurella multocida* A | Lungs | Hubei | + | + |
| *Pasteurella multocida* A | Lungs | Hunan | + | + |
| *Pasteurella multocida* A | Lungs | Sichuan | + | + |
| *Pasteurella multocida* A | Lungs | Sichuan | + | + |
| *Pasteurella multocida* A | Lungs | Guangdong | + | + |
| *Pasteurella multocida* A | Lungs | Guangdong | + | + |
| *Pasteurella multocida* A | Lungs | Hubei | + | + |
| *Pasteurella multocida* A | Lungs | Guangdong | + | + |
| *Pasteurella multocida* A | Lungs | Beijing | + | + |
| *Pasteurella multocida* A | Lungs | Guangdong | + | + |
| *Pasteurella multocida* A | Lungs | Guangdong | + | + |
| *Pasteurella multocida* A | Lungs | Hubei | + | + |
| *Pasteurella multocida* A | Lungs | Guangdong | + | + |
| *Pasteurella multocida* A | Lungs | Guangdong | + | + |
| *Pasteurella multocida* A | Lungs | Beijing | + | + |
| *Pasteurella multocida* A | Lungs | Guangdong | + | + |
| *Pasteurella multocida* A | Lungs | Hubei | + | + |
| *Pasteurella multocida* A | Lungs | Hubei | − | + |
| *Pasteurella multocida* A | Lungs | Hubei | + | + |
| *Pasteurella multocida* A | Lungs | Hubei | + | + |
| *Pasteurella multocida* A | Lungs | Guangdong | + | + |
| *Pasteurella multocida* A | Lungs | Hubei | + | + |
| *Pasteurella multocida* A | Lungs | Hubei | + | + |
| *Pasteurella multocida* A | Lungs | Guangdong | + | + |
| *Pasteurella multocida* A | Lungs | Hubei | + | + |
| *Pasteurella multocida* A | Lungs | Guangdong | + | + |
| *Pasteurella multocida* A | Lungs | Guangdong | + | + |
| *Pasteurella multocida* D | Nasal swabs | Hainan | − | − |
| *Pasteurella multocida* D | Lungs | Guangdong | − | − |
| *Pasteurella multocida* D | Lungs | Guangdong | − | − |
| *Pasteurella multocida* D | Lungs | Hubei | − | − |
| *Pasteurella multocida* D | Lungs | Anhui | − | − |
| *Pasteurella multocida* D | Lungs | Henan | − | − |
| *Pasteurella multocida* D | Lungs | Hubei | − | − |
| *Pasteurella multocida* D | Lungs | Hubei | − | − |
| *Pasteurella multocida* D | Lungs | Hubei | − | − |
| *Pasteurella multocida* F | Lungs | Henan | − | − |

**Note:** Bacterial strain susceptibility for phage PHB02 was assessed using the spotting method (Chen et al., 2018 see Table S1). All *Pasteurella multocida* strains used in this study were isolated from pigs, except for strain HB01, which was isolated from cattle and they were maintained in the Key Laboratory of Agricultural Microbiology, Huazhong Agricultural University. a (+) indicates cleavage, (−) indicates no cleavage; b (+) indicates cleavage, (−) indicates no cleavage.
