# Supplementary material for: Therapeutic Application of Bacteriophage PHB02 and Its Putative Depolymerase Against Pasteurella multocida Capsular Type A in Mice
Source: Front Microbiol. 2018 Aug 7;9:1678. doi: 10.3389/fmicb.2018.01678 (PMC6090149; doi:10.3389/fmicb.2018.01678)
Supplement: Supplementary file 3 [file Table_2.DOCX]

**Table S2. Primers and sequence used in this study**.

| Primers | Primer sequence (5′-3′) | Restriction site | Size (bp) |
| --- | --- | --- | --- |
| *HexA*F1 | GCGTCGACGTGCGGTTTCTGCCAATAGTA | *Sal*I | 517 |
| *HexA*R1 | CGGAATTCCGAGGTTCTATCCCTTTACTG | *Eco*RI |  |
| *HexA*F2 | CGGAATTACAAAAACCCGCTTATCG | *Eco*RI | 504 |
| *HexA*R2 | TCCGAGCTCGGAGGATTCGGCGGAGGA | *Sac*I |  |
