# Supplementary material for: Therapeutic Application of Bacteriophage PHB02 and Its Putative Depolymerase Against Pasteurella multocida Capsular Type A in Mice
Source: Front Microbiol. 2018 Aug 7;9:1678. doi: 10.3389/fmicb.2018.01678 (PMC6090149; doi:10.3389/fmicb.2018.01678)
Supplement: Supplementary file 4 [file Table_3.DOCX]

**Table S3. Infected with *P.multocida* HB03 in mice.**

| Dose of *P.multocida* HB03 (CFU) | The survival rate of mice (mice surviving/ mice in group) |
| --- | --- |
| 10 | 3/3 |
| 20 | 2/3 |
| 40 | 0/3 |
| 80 | 0/3 |
| 160 | 0/3 |
